# Nitrogen cycling in an extreme hyperarid environment inferred from δ^15^N analyses of plants, soils and herbivore diet

**DOI:** 10.1038/srep22226

**Published:** 2016-03-09

**Authors:** Francisca P. Díaz, Matías Frugone, Rodrigo A. Gutiérrez, Claudio Latorre

**Affiliations:** 1 Departamento de Ecología, Pontificia Universidad Católica de Chile, Alameda 340, Santiago, Chile; 2 Institute of Ecology and Biodiversity (IEB), Las Palmeras 3425, Ñuñoa, Santiago, Chile; 3 FONDAP Center for Genome Regulation and Millennium Nucleus Center for Plant Systems and Synthetic Biology. Departamento de Genética Molecular y Microbiología, Pontificia Universidad Católica de Chile, Alameda 340, Santiago, Chile; 4 Laboratorio Internacional de Cambio Global, LINCGlobal PUC-CSIC; 5 Instituto Pirenaico de Ecología, Montañana, España

## Abstract

Climate controls on the nitrogen cycle are suggested by the negative correlation between precipitation and δ^15^N values across different ecosystems. For arid ecosystems this is unclear, as water limitation among other factors can confound this relationship. We measured herbivore feces, foliar and soil δ^15^N and δ^13^C values and chemically characterized soils (pH and elemental composition) along an elevational/climatic gradient in the Atacama Desert, northern Chile. Although very positive δ^15^N values span the entire gradient, soil δ^15^N values show a positive correlation with aridity as expected. In contrast, foliar δ^15^N values and herbivore feces show a hump-shaped relationship with elevation, suggesting that plants are using a different N source, possibly of biotic origin. Thus at the extreme limits of plant life, biotic interactions may be just as important as abiotic processes, such as climate in explaining ecosystem δ^15^N values.

Global data syntheses have demonstrated an inverse relationship between foliar δ^15^N and mean annual precipitation (MAP) and a direct relationship with mean annual temperature (MAT) over a wide variety of ecosystems and indicate that climate is a major driver of the N cycle^1^,^2^,^3^,^4^,^5^,^6^. Positive δ^15^N (6–10%) values are typical of arid regions^7^,^8^ whereas low δ^15^N values (−4–0%) are typical for cold/humid sites and/or those with elevated rainfall^9^,^10^. Such negative correlations between foliar δ^15^N and/or surficial soil δ^15^N and MAP have been described for the United States^11^, South Africa^12^, tropical forests in Hawaii^13^, Australia^14^ and Ethiopia^15^ among other regions.

Nitrogen isotopes integrate multiple ecological inputs and outputs including ammonification, nitrification and denitrification of N in the soil. Fractionation at each step and environmental drivers -such as precipitation- contribute to ecosystem N fluxes^16^,^17^,^18^. Plants take up inorganic N from soils and in some cases (i.e. the Fabaceae) from atmospheric N (N_2_) sources through symbiotic association with nitrogen-fixing bacteria. The impact of MAT on δ^15^N is not as clear. Some studies show no relationship between MAT and δ^15^N^19^, but others suggest that increased MAT correlates with higher δ^15^N values. A recent global syntheses^6^ shows that apart from MAT and MAP, soil concentration of organic carbon and clay also exhibit a strong relationship with soil δ^15^N.

Resolving how climate affects ecosystem N inputs and outputs (thus driving changes in isotope values) is complex because the multiple timescales involved (10^2^–10^3^ years) are not amenable to experimental procedures. Space can be substituted for time, however, and studies across environmental gradients have been used as “natural experiments”^13^. The downside is that these studies often deal with many potential variables that can also affect soils (e.g. substrate, depth, age) and plants (e.g. photosynthetic pathway, N_2_-fixers).

With limited precipitation and human impact, arid landscapes offer one way of disentangling the myriad variables that can affect isotope landscapes^7^,^15^,^20^ although these often exhibit contrasting results^20^. The Atacama Desert is far from human perturbations (such as industrial and agricultural land use) and exhibits pronounced climate gradients over short (<50 km) distances (Fig. 1). It is thus an ideal scenario for testing the direct effects of extreme aridity on the δ^15^N signal and how these changes relate to the nitrogen cycle and climate.

The Atacama is a cold, hyperarid environment, where the lack of rainfall and to a lesser degree, temperature play primary roles in determining the presence of plants^21^. We performed an elevational survey at ~23.5 °S, from Laguna Lejía (~4500 m) to the eastern margin of the Salar de Atacama (near Talabre, at 2700 m) here referred to as the Talabre-Lejía Transect (TLT). Located in the western Andes Cordillera, the TLT runs south of the active Lascar volcano (the last major eruption was in 1993). Most of our soil and plant plots occur on incipient alluvial and colluvium soils derived from volcanic parent material, which at most sites is underlain by the Pliocene Patao ignimbrite^22^. Ignimbrite outcrops are common at our lower elevation sites (from 3300 to 2700 m).

Relative humidity is often ~10% in many areas of the Atacama Desert (with no recorded rainfall in over 30 years at some stations <2000 m). Summer precipitation is of tropical origin (which increases with elevation and decreases with latitude) and this gives way to increased winter precipitation south of 26 °S latitude^23^,^24^.

Along the TLT (4500–2700 m), MAP and MAT range from ~160 mm/yr and 4.2 °C at our upper sites, to ~10 mm/yr and 13 °C at our lower sites^24^,^25^. Both the upper and lower limits of the survey represent extremes in environmental conditions. At the upper limit, vegetation is restricted by extremely low temperatures, wind and snow, whereas at the lower limit it marks the onset of the absolute desert, with almost no precipitation. Moisture from fog is confined to the coastal escarpment^26^.

The interaction of increasing rainfall and decreasing temperature with elevation is closely tracked by vegetation richness and %cover, which increases with elevation, peaks near the center of the gradient and decreases towards higher elevations. Several vegetation belts or zones are easily recognized based on overall plant physiognomy^27^,^28^,^29^. The “Andean steppe” dominates between 4500–4000 m characterized by perennial bunch grasses including *Jarava frigida, Calamagrostis crispa* and cushion plants and sub-shrubs such as *Mulinum crassifolium* (Apiaceae). The “puna” (or tolar) forms between 4000–3300 m and is dominated mostly by shrubs, sub-shrubs and perennial herbs such as *Parastrephia quadrangularis, Baccharis tola* (both Asteraceae) and *Junellia seriphioides* (Verbenaceae). Rainy summers are followed by large annual blooms that include *Lupinus subinflatus* (Fabaceae), *Montiopsis* sp. (Montiaceae) and several C_4_ annual grasses (*Munroa, Bouteloua, Aristida*). The lowermost vegetation belt is the “prepuna” from 3300 to 2700 m. Only cushion cacti *Maihueniopsis camachoi* and the C_4_ sub-shrub *Atriplex imbricata* (Amaranthaceae) and the sub-shrub *Tiquilia atacamensis* (Boraginaceae) can withstand the drier conditions. A few annuals such as *Aristida adscensionis* (Poaceae) and *Exodeconus integrifolius* (Solanaceae) can flourish here after very wet summers (March-April).

We surveyed and collected plants, surface soil samples and herbivore feces to assess impacts on diet along the 1800 m elevational gradient (TLT) that ranges from the extreme hyperarid conditions at 2700 meters above sea level (m) to extremely cold conditions at 4500 m. We obtained soil physical (%sand, %clay, %silt) and chemical properties, including total N, NO_3_, NH_4_, pH, organic matter (OM), macronutrients (P, C, S, K), SAR (sodium adsorption ratio) and analyzed our soil samples for δ^15^N to better understand the factors that could be behind N cycling as well as soil development along this climatic/altitudinal gradient.

We describe the relationship between soils, foliar samples and herbivore feces with climate across this gradient and construct the isotope landscape for this extreme environment. We evaluate the mechanisms proposed to explain these relationships, divided into those that focus on abiotic factors (i.e. climate, pH) and those that focus on biotic factors (i.e. diversity, presence of N_2_-fixers and how these relate to microorganisms). Finally, we compare soil, plant and herbivore feces δ^15^N values to infer how N acquisition occurs in plants from these extreme environments.

## Results

### Plant and soil descriptions

MAP, MAT and therefore aridity are mainly a function of elevation in this area of the Atacama. All of these variables co-vary and we were unable to discern which had the most explanatory power. Hence, we present our results in relation to elevation in the understanding that this co-varies positively with MAP and aridity (R^2^ = 0.93, p < 0.001) and negatively co-varies with MAT (R^2^ = −0.99, p < 0.001). Plant species richness and percent relative cover exhibit a “hump-shaped” curve with elevation (Fig. 2). The calculated *De Martonne* aridity index (0–5 hyperarid, 5–10 arid and 10–20 semiarid)^30^ varied between 11.4 at 4500 m and 0.5 at 2700 m (Table 1). Zones characterized as hyperarid by this index are approximately those between 2700 and 3900 m.

A pronounced pH gradient occurs along the TLT, with acidic soils occurring at high elevations (pH 5.23) and alkaline soils at lower elevations (pH 8.54) (Table 1). Soil pH shows significant correlations with δ^15^N, δ^13^C, aridity, mean C (mg/kg) and some macronutrients (N, P, S, K) (Table 2). The soil physical composition is mainly sand ~81%, followed by silt ~12% and finally, clay ~7%. A negative correlation (R^2^ = −0.66, p < 0.005) occurs between % clay and the aridity index (a higher index means less aridity). Total nitrogen was low throughout the environmental gradient (0.26–0.60 mg/g) and shows no significant correlation with aridity (R^2^ = 0.19, p > 0.05) (Table 2). Other soil parameters (total N, NO_3_, C and P) correlate with biotic factors and do not correlate with aridity. These show increases at mid-elevations, where temperature is not as cold as at higher elevations and rainfall is not as dry as at lower elevations. Hence, plant diversity, plant cover and organic matter (OM) are highest at mid-elevations along our gradient (Table 2 and Fig. 2). P increases with MAP, whereas K decreases. A high sodium adsorption ratio (SAR) implies poorly irrigated soils and as expected, correlates positively with aridity.

### Soil isotopes

The isotopic δ^15^N_soil_ values range from 3.3% to 12.2% with a δ^15^N_soil_ mean of 8.2% (n = 40) (Table 3 shows the mean site δ^15^N_soil_ values, the entire list of 40 surficial soil samples can be found in Supplementary Table 1). Regression analysis shows that δ^15^N_soil_ values are strongly dependent on elevation, which explains 72% of the variance (Fig. 3, R^2^ = 0.72, *p* < 0.001).

No apparent differences were observed between soil samples taken in different years after the rainy season (April 2011, 2012 and 2013) (Supplementary Table 1). We also compared how δ^15^N_soil_ values changes with soil depth (0, 25 and 50 cm) at three representative sites (2900, 3600 and 4300 m) and found no apparent differences (Supplementary Table 3).

### Foliar isotopes

δ^15^N_foliar_ values range from **−**2.0 to 8.8% and the δ^13^C_foliar_ ranges from −27.1% to −11.5% (n = 66, Table 4, Fig. 3). Mean site δ^15^N_foliar_ shows a significant inverse correlation (R ^2^ = 0.48, p* *<* *0.001) with elevation, although a second-order “hump-shaped” polynomial does a slightly better job at explaining the variance observed (R^2^ = 0.58, *p *<* *0.001, Fig. 3). Individual plant δ^15^N_foliar_ values within each site are often highly variable with standard deviations ranging from 0.4 to 3.1%. Most of this variation could be due to different species being sampled (Supplementary Table 2). We compared seed and stem δ^15^N values for the cushion cactus *Maihueniopsis camachoi* and found only slight variation (~1%) across these different plant tissues (Supplementary Table 2).

Among different plant species, *Jarava frigida* show the lowest average δ^15^N_foliar_ value (1.3%) and *Tiquilia atacamensis* the highest (6.5%). The average δ^15^N_foliar_ value is 4.4% for *Baccharis tola,* 4.4% for *Parastrephia quadrangularis,* 6.0% for *Maihueniopsis camachoi* and 6.2% for *Atriplex imbricata. M*ean δ^15^N_foliar_ per site shows a hump-shaped relationship with elevation, from 4500 to 2700 m (Figs 3 and 4).

### Herbivore feces isotopes

Herbivores feces, mainly rodents (N = 10) and camelids (N = 9), show a humpback relationship with elevation (R^2^ = 0.67, p* *<* *0.001) with no differential elevational responses across the different species sampled. The δ^15^N values (Table 5) ranges from 2.1% to 8.3% (Fig. 4). Plant δ^15^N values predict 53% (R^2^ = 0.53, p < 0.005) of the variance observed in herbivore δ^15^N. Although plants and herbivores show similar signals (Fig. 4), at mid-elevations, herbivores (as expected by trophic enrichment) tend to be enriched in ^15^N by ~1% compared to plants. At the extremes of our gradient, however, plants are enriched compared to herbivores, suggesting that herbivores at these elevations are mixing or selecting their plant sources from other local sources (such as a spring or deep canyon).

## Discussion

Five different mechanisms have been proposed to explain how soil δ^15^N values relate to aridity or climate. These include: (1) high MAP increases soil organic matter which becomes depleted in ^15^N in response to fractionation during mineralization^31^; (2) increase in the discrimination of ^15^N with increasing N soil reserves^17^; (3) elevated temperatures in arid and hyper-arid environments can trigger increased N volatilization and the preferential removal of ^14^N^32^; (4) changes in the relative importance of within-system cycling versus inputs/outputs or the “openness” of the N cycle^1^,^13^; and (5) the relationship between soil δ^15^N and climate is indirect and mediated through climatic effects on soil C concentration and clay amount^6^.

All of our sites exhibited positive δ^15^N_soil_ values (1.9% to 11.6%%), indicating the preferential removal of ^14^N from soils, coupled with overall lack of organic matter. The large ~10% variation is unusual and appears to be related to the extreme hyperaridity of our study system. As expected, the most positive values appear at the driest sites (~2700 m) and soil % N does not change across the TLT (Table 3). Because the organic matter and N content in our sites do not correlate with δ^15^N_soil_ values (Table 2), the aforementioned mechanisms (1) and (2) are unlikely to explain our observations.

Mechanisms (3), (4) and (5) are more coherent with our results. Mechanism (3) could explain the very positive values found in the driest sites which may result from high N volatilization during elevated diurnal temperatures and could be associated with very alkaline soils (~pH = 9)^33^. However, TLT δ^15^N_soil_ values may also increase due to reduced plant N demand at the driest sites (in this case water, rather than N is limiting), forming large pools of inorganic N (mostly nitrate) in the soil subject to further leaching or ammonia volatilization as suggested by mechanism (4). Plant N demands increase with rainfall, creating a larger soil organic N pool. The retention of organic N, instead of inorganic labile pools, reduces the N outputs and increases within-system cycling (i.e. decreasing cycle “openness”). Although leaching would increase compared to the drier sites, organic N is less labile. Increased aridity, salinity and extreme pH decrease the proportional flux of ecosystem N into organic pools and increase the inorganic N pool and outputs. Since nearly all N transformation processes fractionate preferentially for ^14^N^16^, cycle “openness” drives ecosystem N towards ^15^N-enrichment (relative to atmospheric N_2_) in the driest sites. Finally, % clay and carbon (mechanism 5) could explain in part some of the variability. Although soil C concentration exhibits little variation across the gradient (0.89 to 3.33 mg/kg), a significant correlation (R^2^ = −0.53, p < 0.05) with δ^15^N_soil_ values does occur as expected. Percent clay in our soils varies from 3.3 to 11.9% and the correlation with δ^15^N_soil_ values is significant (R^2^ = 0.67, p < 0.005) yet the relation between MAP and % clay is actually inverse (R^2^ = −0.65, p < 0.005) as the amount of clay decreases with elevation. This implies that although % clay is a contributing factor, precipitation and the indirect effect as observed in global climate gradients^6^ may not be the actual mechanism in our gradient from the Atacama. Furthermore, our soil δ^15^N samples exhibit stronger direct correlations with climatic variables (MAP and MAT) than with C or % clay (Table 2).

Other drivers besides climate have been proposed to explain δ^15^N_soil_ variations including soil depth^34^, age^2^ and the presence of N_2_ fixers^35^, among others. Soil depth would not affect our results as all samples were taken from the first 5 cm and we further tested for variations in δ^15^N_soil_ with depth (0, 25 and 50 cm) at three sites and no differences were found (Supplementary Table 3). A few N_2_ fixers (*Adesmia* spp., *Lupinus* spp. and *Hoffmannseggia doelli*) are found along the TLT. These N_2_ fixers are characterized by comparatively low δ^15^N values (close to atmospheric N_2_ ~ 0%)^36^. Some of our sites were dominated by *Lupinus* spp. (*L. subinflatus* and *L. oreophilus*) during wet years, but the presence of these plants was not reflected in our δ^15^N_soil_ values, which are highly enriched compared to atmospheric N_2_.

Ewing and collaborators^37^ proposed a transformation in the N cycle from arid to hyperarid sites where soil N loss decreases to be almost negligible. They described two modes of N accumulation. In semiarid environments, total soil N increases with rainfall and is mostly organic. In arid to hyperarid sites total soil N increases with aridity and is mostly inorganic. We thus expected to find more N accumulated in the soils at our most arid sites. We also expected N to increase at the lower sites as the C/N ratio decreased and biological uptake of N ceased. Surprisingly, we found that although plant cover and total organic matter content decreases at the extremes of our survey, total soil N did not change despite a relative increase in total soil C with elevation (Table 3).

The mean soil δ^15^N value from all sites (8.2 ± 1.8%) concurs with what was expected for such an hyperarid area as extrapolated from a global dataset^6^. Within the gradient, we found an overall ~3% shift that correlates with an order of magnitude shift in MAP, which is more pronounced than the expected ~2% shift inferred from the global dataset^6^. We observed a linear relationship between elevation and soil δ^15^N values (R^2^ = 0.72, p < 0.001), and a hump-shaped curve between elevation and foliar δ^15^N values (R^2^ = 0.58, p < 0.001), (Figs 3 and 4). A hump-shaped second order polynomial is indeed a better fit than a first-order model to our foliar samples (p < 0.05).

These results imply that both biotic (e.g. plant cover, richness) and abiotic (climate gradients) factors constitute possible explanations for the isotopic properties observed. Indeed, δ^15^N_foliar_ and δ^15^N_herbivore_ exhibit a hump-shaped relationship with elevation (Fig. 4) which points to different drivers below and above ~3300 m. For sites >3300 m, foliar δ^15^N appears to be coupled with soil δ^15^N, but <3300 m (where vegetation cover falls to almost zero), these values are much less positive (~4%) than their corresponding soil δ^15^N values (Fig. 3). This apparent “decoupling” could be due to short-term plant δ^15^N dynamics of N cycling versus the long-term dynamics of soil δ^15^N values^38^.

To evaluate if the soil and plant values are effectively coupled or decoupled along the different parts of the gradient or across vegetation belts, we normalized and divided the data into three vegetation belts (prepuna, puna and steppe) (Supplementary Fig. 2). A t-test comparing soils and plants from each vegetation belt reveals no significant differences (p > 0.05) although plants fit a “hump-shaped” distribution better than our soil samples, which are clearly linear with elevation. Nevertheless, these results could be more robust as the number of data within each class is still low, an important prospect for future studies.

A recent study in arid grasslands from China^20^ also described a hump-shaped curve between plant δ^15^N and aridity, similar to our results but with a different threshold. In that study^20^ the tipping point occurs at sites with less than ~200 mm/yr of rainfall. Yet in our study, all sites are below 200 mm and the threshold is closer to 50 mm/yr (~3500 m). They point to a change in net plant N accumulation relative to gaseous N losses (volatilization) as the primary cause in determining the negative relationship between aridity and δ^15^N at the drier sites. The implication is that the net ecosystem N retention rate increases with aridity. This clearly was not the case for the Atacama as soil total N (mg/kg) does not correlate significantly with elevation, MAP or MAT (Table 2).

In animal tissues, δ^15^N values are derived from diet, climate and/or physiology, but there is some disagreement regarding which of these factors is (are) the main driver(s) in the observed ^15^N abundance across environments or taxa^39^,^40^,^41^. It has also long been recognized that δ^15^N in animal tissues and feces are enriched compared to their diet (~3%)^39^,^42^,^43^. Our herbivore feces do not show any consistent ^15^N enrichment compared to plants for those same elevations along our TLT (Fig. 4). In fact, feces ^15^N abundance is overall very similar to the plants at the same elevations and displays a hump-shaped relationship when plotted against elevation, MAP, or MAT (Fig. 4). These results suggest that herbivores feces from the Atacama have an important component of non-digested plants, as seen by the presence of well-preserved fibers and plant remains in rodent feces^44^.

As expected, there is an overall strong negative correlation between the aridity index (which increases with decreasing “aridity”) and soil δ^15^N values (R^2^ = −0.71, p < 0.001) (Fig. 3 and Table 2) and an overall positive correlation in δ^15^N values between soil-plants-herbivores, as plants provide the only exogenous source of N to herbivores^41^,^45^. This relationship breaks down, however, between 3200 and 2700 m, where total vegetation cover drops to <1%. Thus, plants and feces show an enrichment in ^15^N with elevation from 2700 to 3500 m and a depletion in ^15^N with elevation from 3500 to 4000 m (Fig. 4). These results suggest that different drivers for the N isotope natural abundance appear to be at work in the Atacama at different elevations and that feces δ^15^N values are directly established by diet and do not appear to be further enriched by animal metabolism. Previous research has also pointed out that in hyperarid environments, the typical positive relationship between aridity and δ^15^N may not be always accurate^41^,^46^.

We speculate that perhaps the role of microorganisms at such hyperarid sites in the Atacama could also be an important factor, either as free-living soil organisms or in association with plants^47^. Microorganisms are clearly important in fixing atmospheric N_2_ and recycling organic N from leaf litter, processes that are known to deplete ^15^N absorbed by plants and could explain the differences between soil δ^15^N and foliar δ^15^N at our lower elevation sites. Hence, plants could survive above micro-nutrient “islands” with different N sources compared to the surrounding soils in such an extreme environment.

Finally, the extreme positive values seen in our results can help shed further light on the mechanisms underlying the relationship between climate and δ^15^N. The soil δ^15^N pattern that occurs in our gradient appears to favor two different but complimentary hypotheses. As expected, soil δ^15^N values are correlated with elevation and climate, especially above 3300 m. As the driest desert of the world, water is the most important factor controlling plant cover and even diversity. The interplay between precipitation and temperature, however, regulates the presence of vegetation at higher elevations. In contrast, high diurnal temperatures and elevated pH at our most arid sites (~2700 m) would promote ammonium volatilization. Coupled with a more “openness” of the N cycle, this would further increase the loss of labile inorganic N pools through volatilization.

In contrast, the hump-shaped foliar and herbivore feces δ^15^N relationship with elevation points to different drivers along the gradient. Above 3300 m, foliar δ^15^N values are coupled with soil δ^15^N values and are likely regulated by climate and other abiotic factors. In contrast, below 3300 m foliar δ^15^N values become apparently “decoupled” from their corresponding soil δ^15^N values (Supplementary Fig. 2) indicating biotic factors are more prevalent. Our results suggest that greater attention should be devoted to understanding the role of the microbiome in these extreme environments, where they could be important drivers of soil N availability.

## Methods

**Figure 1:**
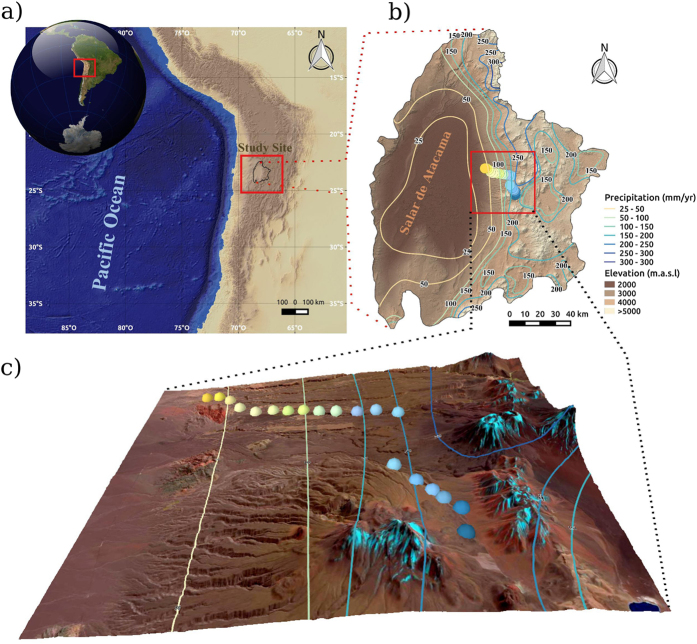
Study site, climate and sampling sites in northern Chile. Regional context of northern Chile showing location of the Salar de Atacama and adjacent Andes (right inset) and a digital elevation model indicating where our sampling sites (lower inset, colored dots) are along the Talabre-Lejía Transect (TLT). Isohyets are in the same corresponding color as the respective sampling sites. The software used to create the map was QGIS 2.10 with Openlayer plugin, STRM30^53^,^54^ elevation model (Data: SIO, NOAA, U.S. Navy, NGA, GEBCO) and Landsat 8 Satellite image (Data available from the U.S. Geological Survey).

**Figure 2:**
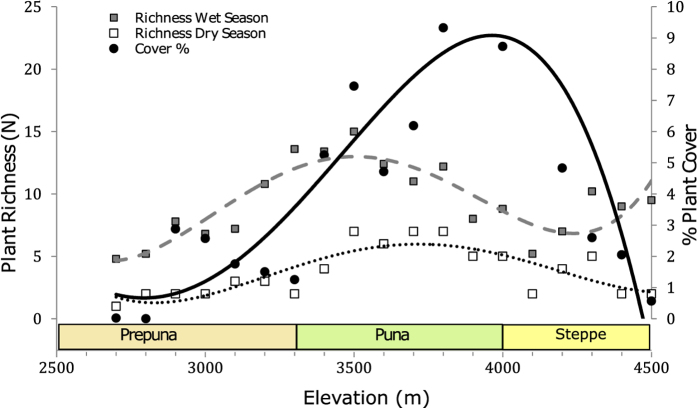
Average % plant cover and plant species richness for the Talabre-Lejía Transect (TLT). Plant species richness and plant cover were estimated using the McAuliffe log-series survey method^48^ using two replicate plots of 250 m^2^ each. Circles (•) represent the plant cover % during the wet season. Squares represent richness or number of species during the wet season (▪) in April, and dry season (□) in July. Best-fits are indicated by polynomial regressions and are plotted to show overall trends in the data.

**Figure 3:**
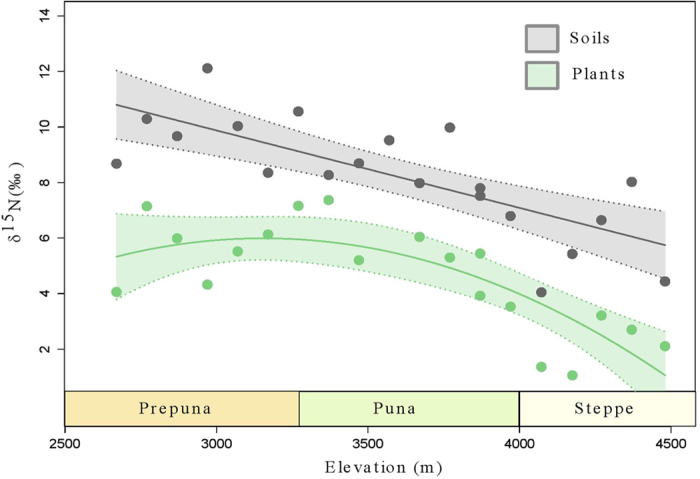
The relationship between mean soil δ^15^N and mean foliar δ^15^N. Elevation across the transect (TLT), versus mean soil δ^15^N values (20 averages obtained from 40 samples) and mean foliar δ^15^N values (19 averages obtained from 66 samples). Soils are fit with a linear regression model, R^2^_bootstrapping_ = 0.73 ± 0.20, *p *<* *0.001. A second-order polynomial regression was fitted to describe the relationship between foliar δ^15^N and elevation (R^2^_bootstrapping_ = 0.56 ± 0.21, *p*_*c1*_* *<* *0.005, *p*_*c2*_* *<* *0.05). Colored shadowing indicates the 95% confidence intervals.

**Figure 4:**
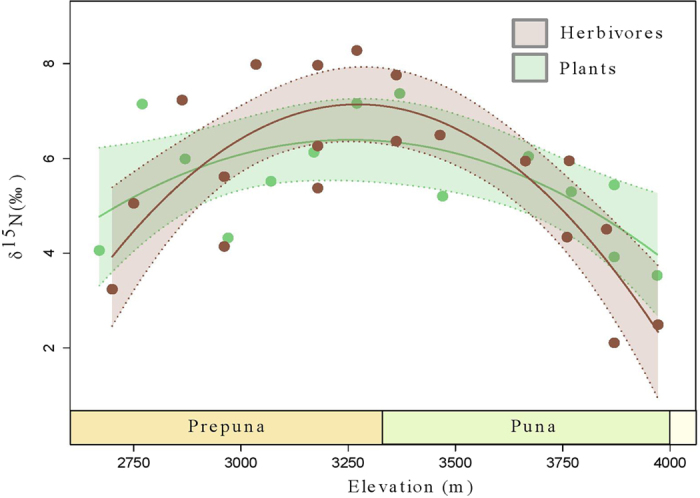
The relationship between mean foliar δ^15^N and mean herbivore feces δ^15^N. Elevation across the transect (TLT) versus mean foliar δ^15^N values (4000–2700 m) and herbivore feces δ^15^N values. The fitted curves are second order polynomial regressions (plants: R^2^_bootstrapping_ = 0.60 ± 0.28, *p*_*c1*_* *<* *0.18, *p*_*c2*_* *<* *0.001; herbivores: R^2^_bootstrapping_ = 0.67 ± 0.23, *p*_*c1*_* *<* *0.05, *p*_*c2*_* *<* *0.001). Colored shadowing indicates 95% confidence intervals.

We characterized 20 sites every 100 m of altitude (Fig. 1 and Table 1) during four consecutive years (2011–2014), in April (after the rains, during the growing season). Maximum elevation was 4500 m (MAP ~ 160 mm) and the lowest elevation was at 2700 m (MAP ~ 10 mm) (Fig. 1). The *De Martonne* aridity index was calculated at each site as described in previous reports^30^ (Table 1). Plant richness and plant cover were estimated using the McAuliffe log-series survey^48^ with two plots of 250 m^2^ for each of the 20 sites along the TLT (Fig. 2).

We analyzed two independently collected soil samples from each site. We collected each isotopic soil sample by removing the top 1 cm of surface soil and collecting the next five centimeters (from five different soils in each site). Samples were taken to the laboratory in sealed plastic bags, dried at 50 °C, and sieved and inspected under a binocular microscope to remove visible plant remains such as rootlets, before the δ^15^N and δ^13^C isotopic analyses. Table 3 shows mean values and the entire list of 40 soil samples can be found in Supplementary Table 1. Foliar samples were collected from various individual ramets of each species and homogenized for total C, N and δ^13^C, δ^15^N analyses (Table 4, mean values). As it is impossible to collect the same plant species across the entire gradient, six species were selected for isotope analyses (*Baccharis tola, Jarava frigida, Maihueniopsis camachoi, Parastrephia quadrangularis, Tiquilia atacamensis* and *Atriplex imbricata*) based on their widespread distribution within the Atacama gradient. The relative % plant cover of these species was at least 50% at each site. Leaves, fruits or seeds were sampled from 66 specimens and analyzed for total C, N and δ^13^C, δ^15^N (Supplementary Table 2). The plants were collected, pressed and dried in the field, and later placed in a drying oven at 50 °C in the laboratory (the entire list of 66 samples can be found as Supplementary Table 2). We also collected and analyzed 19 feces samples collected along the TLT during April 2011–2014 (Table 5). Rodents (*Abrocoma* and *Phyllotis)* are the most common taxa sampled (N = 10), but we also collected camelid feces (N = 9).

δ^15^N was standardized with N_2_-Air and δ^13^C values to Vienna Pee Dee Belemnite (VPDB). The δ values were measured in units of per mil (%). A total of 36 soil and 50 foliar samples were submitted to the Cornell University Stable Isotope Laboratory (COIL) and analyzed on a Thermo Delta V Advantage isotope ratio mass spectrometer (IRMS) coupled with a NC2500 Elemental Analyzer. Herbivore feces (n = 19) and additional soil (14) and foliar (16) samples were measured at the Laboratory for Biogeochemistry and Applied Stable Isotopes (LABASI) of the Departamento de Ecología, Pontificia Universidad Católica de Chile using a Thermo Delta V Advantage IRMS coupled with a Flash2000 Elemental Analyzer (see Supplementary Tables 1 and 2). Overall, agreement between these two labs was tested by same sample comparisons and reproducibility was within the analytical error of these instruments (±0.2%).

Soil samples for chemical analysis parameters were also collected from all stations along our survey during April 2012, 2013 and 2014. These data are used to calculate the correlation matrix (Table 2) as an average from these three years. Soil composition can change across the landscape, and also within sites, according to soil moisture, soil type, topography, aspect or vegetation, particularly in arid ecosystems, thus generating a heterogeneous biogeochemical landscape^49^. To handle this heterogeneity, the soil samples from each station consisted of the soil collected and mixed from 10 randomly placed quadrants (diameter: 15 cm, depth: 5 cm) (Supplementary Table 1).

Soil texture and chemical analysis was carried out by the Laboratorio Agroanalisis UC, Facultad de Agronomía e Ingeniería Forestal, Pontificia Universidad Católica de Chile, according to the methods established by the CNA of the Chilean Society of Soil Science^50^. Analyses included grain size, elemental composition, and soil pH, among others (see Table 2).

### Statistical Analysis

Simple linear models were used to study correlations and variation in δ^15^N and δ^13^C values. The soil and climatic parameters (pH, N (mg/g), C (mg/kg), MAP and MAT among others) were correlated with Pearson’s correlation coefficient (Table 2). To analyze the coupling of the soil and foliar samples across the different vegetation belts or elevation we normalized each dataset, which are then graphed as box plots (Supplementary Fig. 2). All analyses, charts and maps were performed in the R Programming Language and Quantum GIS software^51^,^52^.

## Additional Information

**How to cite this article**: Díaz, F. P. *et al*. Nitrogen cycling in an extreme hyperarid environment inferred from δ^15^N analyses of plants, soils and herbivore diet. *Sci. Rep.* **6**, 22226; doi: 10.1038/srep22226 (2016).

## Supplementary Material

Supplementary Information

## Figures and Tables

**Table 1:** Geospatial, soil and climatic data for all sites sampled.

Site	Altitude (m asl)	Latitude °S	Longitude °W	Soil Regolith	Slope	Aspect	pH Susp.	MAP*	MAT**	Aridity De Martonne Index
TLT01	4480	−23.50305	−67.72371	Alluvial, ignimbrite	40 °	N	5.23	161.9	4.2	11.4
TLT02	4370	−23.45127	−67.77322	Aeolian, volcanic	1 °	NW	5.31	142.4	4.5	9.8
TLT03	4270	−23.4323	−67.77125	Sandy alluvial, ignimbrite	5 °	W	5.73	125.9	4.8	8.5
TLT04	4174	−23.42283	−67.78008	Colluvium, volcanic	28 °	W 10° S	5.14	111.4	5.3	7.3
TLT05	4072	−23.41518	−67.78575	Colluvium, volcanic	17 °	N 60° E	5.37	97.4	6.1	6.0
TLT06	3970	−23.40508	−67.79452	Sandy, colluvium	11 °	N 70° W	5.54	85.0	6.5	5.2
TLT07	3870	−23.38819	−67.80757	Sandy alluvial, volcanic	5 °	W	5.68	74.2	6.9	4.4
TLT08	3870	−23.32856	−67.79890	Sandy alluvial, volcanic	5 °	N 60° E	5.77	75.1	6.9	4.4
TLT09	3770	−23.32218	−67.81620	Alluvial, volcanic	6 °	W	6.63	65.2	7.5	3.7
TLT10	3670	−23.32268	−67,83180	Alluvial sandy, ignimbrite (thick)	9 °	S	6.07	56.4	8.0	3.1
TLT11	3570	−23.31946	−67.84906	Sandy alluvial, volcanic (thick)	10 °	W	6.38	48.6	8.4	2.6
TLT12	3470	−23.31766	−67.86342	Alluvial, volcanic	3 °	N 60° W	7.18	41.7	9.0	2.2
TLT13	3370	−23.31377	−67.87685	Fluvial, sandy, volcanic	3 °	N 60° W	8.11	35.6	9.6	1.8
TLT14	3270	−23.31291	−−67.89014	Alluvial, ignimbrite	6 °	N 70 ° W	7.30	30.2	9.9	1.5
TLT15	3170	−23.31010	−67.90332	Alluvial, ignimbrite	10 °	N 30 °E	7.50	25.6	10.6	1.2
TLT16	3070	−23.31005	−67.91876	Alluvial, ignimbrite	5 °	N 20 ° E	8.09	21.5	10.9	1.0
TLT17	2970	−23.30211	−67.93427	Alluvial, ignimbrite	5 °	N 30 °E	8.26	18.0	11.4	0.8
TLT18	2870	−23.28868	−67.94587	Late Quaternary fluvial terrace	0 °		8.44	15.0	11.8	0.7
TLT19	2770	−23.28113	−67.95757	Alluvial, ignimbrite	5 °	N 20 ° W	8.58	12.4	12.2	0.6
TLT20	2670	−23.28023	−67.96940	Alluvial, ignimbrite	5 °	W	8.54	10.2	12.6	0.5

*MAP based on data from the Direccion General de Aguas (see Houston^24^).

**MAT extracted from Hijmans *et al*.^25^ (Data range 1950–2000).

**Table 2:** Correlation matrix between soil variables.

	Mean δ^15^N	Mean δ^13^C	MAP	MAT	Aridity index	%Clay	%Silt	%Sand	Total N mg/g	NO_3_mg/kg	NH_4_mg/kg	pH susp.	%OM	C mg/kg	P mg/kg	S mg/kg	K mg/kg
Mean δ^13^C	0.69**																
MAP	−0.71***	−0.80***															
MAT	0.72***	0.91**	−0.96***														
Aridity index	−0.71***	−0.77***	0.99***	−0.94***													
%Clay	0.67**	0.51*	−0.65**	0.60**	−0.66**												
%Silt	0.29	−0.01	−0.43	0.28	−0.45	0.66**											
%Sand	−0.51*	−0.26	0.58**	−0.47*	0.60**	−0.90***	−0.92***										
Total N mg/g	−0.45	−0.52*	0.24	−0.43	0.19	−0.16	0.12	0.01									
NO_3_ mg/kg	0.17	0.61**	−0.26	0.42	−0.21	0.08	−0.45	0.22	−0.40								
NH_4_ mg/kg	−0.32	−0.39	0.44	−0.50*	0.44	−0.31	−0.30	0.33	0.35	−0.11							
pH susp.	0.76***	0.88***	−0.88***	0.95***	−0.85***	0.68**	0.31	−0.53*	−0.60**	0.46*	−0.54*						
%OM	−0.25	−0.46*	0.09	−0.33	0.02	0.05	0.35	−0.23	0.89***	−0.54*	0.32	−0.49*					
C mg/kg	−0.53*	−0.61**	0.37	−0.52*	0.32	−0.25	−0.01	0.14	0.81***	−0.24	0.35	−0.62**	0.76***				
P mg/kg	−0.70***	−0.80***	0.66**	−0.81***	0.62**	−0.46*	−0.06	0.27	0.80***	−0.54*	0.50*	−0.90***	0.74***	0.73***			
S mg/kg	0.48*	0.73***	−0.45	0.56*	−0.42	0.43	−0.08	−0.17	−0.36	0.52*	−0.21	0.53*	−0.28	−0.32	−0.51*		
K mg/kg	0.77***	0.82***	−0.81***	0.83***	−0.80***	0.87***	0.41	−0.68**	−0.40	0.37	−0.34	0.89***	−0.23	−0.42	−0.73***	0.64**	
SAR	0.52*	0.88***	−0.54*	0.68**	−0.50*	0.34	−0.23	−0.04	−0.54*	0.68**	−0.33	0.66**	−0.49*	−0.49*	−0.65**	0.90***	0.64**

(***): p < 0.001; (**): p < 0.01; (*): p < 0.05; ( ): not significative.

**Table 3:** Mean soil N and C isotopic values and biogeochemical parameters.

Mean soils		Mean δ^15^N per site	Mean δ^13^C per site	%N	%C	C/N
Site	Elevation					
TLT01	4480	5.9	−23.3	0.01	0.16	16.0
TLT02	4370	7.6	−24.3	0.02	0.19	9.5
TLT03	4270	6.6	−24.4	0.02	0.17	8.5
TLT04	4174	5.9	−23.8	0.03	0.29	9.7
TLT05	4072	4.0	−22.6	0.02	0.23	11.5
TLT06	3970	6.8	−22.2	0.02	0.29	14.5
TLT07	3870	7.7	−22.5	0.03	0.35	11.7
TLT08	3870	7.5	−22.5	0.02	0.32	16.0
TLT09	3770	9.1	−22.7	0.03	0.26	8.7
TLT10	3670	8.0	−22.0	0.02	0.19	9.5
TLT11	3570	9.5	−21.1	0.02	0.15	7.5
TLT12	3470	8.7	−19.6	0.02	0.17	8.5
TLT13	3370	6.6	−21.5	0.03	0.32	10.7
TLT14	3270	10.6	−17.9	0.03	0.21	7.0
TLT15	3170	8.4	−19.0	0.02	0.14	7.0
TLT16	3070	10.0	−18.5	0.01	0.10	10.0
TLT17	2970	10.0	−14.4	0.02	0.13	6.5
TLT18	2870	9.7	−18.3	0.01	0.10	10.0
TLT19	2770	11.1	−13.2	0.01	0.13	13.0
TLT20	2670	8.7	−12.4	0.01	0.09	9.0

Data were obtained from 40 different soil samples (Supplementary Table 1).

**Table 4:** Mean foliar N and C isotopic values.

Site	Elevation	Mean δ^15^N per site	SD	Mean δ^13^C per site	SD
TLT01	4480	2.1	0.65	−24.6	0.79
TLT02	4370	2.7	2.90	−24.7	0.58
TLT03	4270	3.2	0.42	−25.5	2.12
TLT04	4174	1.1	1.88	−24.2	1.24
TLT05	4072	1.4	3.00	−24.4	1.10
TLT06	3970	3.5	2.91	−20.9	4.67
TLT07	3870	3.9	3.12	−20.4	4.21
TLT08	3870	5.4	2.66	−19.2	4.59
TLT09	3770	5.3	0	−26.4	0
TLT10	3670	6.0	1.40	−18.5	5.42
TLT12	3470	5.2	2.15	−17.6	4.76
TLT13	3370	7.4	1.77	−14.0	1.97
TLT14	3270	7.2	0.82	−16.2	5.50
TLT15	3170	6.1	1.27	−19.0	6.63
TLT16	3070	5.5	1.57	−13.7	2.05
TLT17	2970	4.3	1.33	−13.9	2.25
TLT18	2870	6.0	0.55	−19.5	6.64
TLT19	2770	7.2	0	−12.5	0
TLT20	2670	4.1	0.47	−18.5	7.85

SD: Standard deviation Data were obtained from 66 different foliar samples (See details in Supplementary Table 2).

**Table 5:** N and C isotopic values from herbivore feces.

Elevation	δ^15^N	δ^13^C	%N	%C	Agent
3972	2.5	−23.2	4.6	39.2	Camelid
3870	2.1	−27.0	1.3	38.0	Camelid
3852	4.5	−22.7	22.2	39.1	Rodent, Phyllotis
3765	6.0	−25.1	4.6	42.9	Camelid
3760	4.3	−24.6	2.4	48.2	Rodent, Abrocoma
3663	6.0	−21.5	4.4	39.9	Rodent, Abrocoma
3464	6.5	−20.7	7.0	41.5	Camelid
3362	6.4	−21.6	10.3	29.6	Rodent, Abrocoma
3362	7.8	−20.7	7.1	43.4	Camelid
3270	8.3	−23.1	2.5	25.7	Rodent, Lagidium
3179	8.0	−18.4	7.3	30.2	Rodent, Abrocoma
3179	5.4	−24.5	5.9	39.2	Camelid
3179	6.3	−19.5	5.7	29.1	Camelid
3035	8.0	−20.7	8.3	23.5	Rodent, Abrocoma
2961	4.1	−23.1	4.4	43.8	Camelid
2961	5.6	−23.3	4.8	33.9	Camelid
2863	7.2	−23.2	12.3	33.4	Rodent, Abrocoma
2750	5.1	−18.2	7.0	26.0	Rodent, Abrocoma
2700	3.2	−19.5	2.9	10.9	Rodent, Abrocoma

Feces from Talabre-Lejía Transect (TLT) All these samples were analized at the LABASI (Isotope laboratory).
