# Supplementary material for: Nitrogen cycling in an extreme hyperarid environment inferred from δ15N analyses of plants, soils and herbivore diet
Source: Sci Rep. 2016 Mar 9;6:22226. doi: 10.1038/srep22226 (PMC4783660; doi:10.1038/srep22226)
Supplement: Supplementary Information [file srep22226-s1.doc]

**Nitrogen cycling in an extreme hyperarid environment inferred from δ^15^N analyses of plants, soils and herbivore diet**

Francisca P. Díaz, Matías Frugone, Rodrigo A. Gutiérrez & Claudio Latorre.

SUPPLEMENTARY INFORMATION

Carbon (C) is another important macronutrient in soils and the carbon isotopic composition (δ^13^C) changes depending on what plants are growing and the inorganic input product of the reaction between the atmospheric CO_2_ (-9 ‰) and water. The relationship between climate and δ^13^C in plants is better understood than for δ^15^N. First, it depends on the photosynthetic pathway utilized: C_3_ (ca. -26 to -19 ‰), C_4_ (-14 to -12 ‰) or CAM (variable values between C_3_ and C_4_). Second, δ^13^C values in C_3_ plants increase with water use efficiency as stomatal closure becomes more frequent to avoid desiccation.

Our results show that mean δ^13^C_soil_ has an inverse relationship with elevation (Supplementary Fig. 1a). The δ^13^C_soil_ values ranges from -24.4‰ to -14.8‰ (Table 2). A positive linear correlation between δ^15^N and δ^13^C exist (R^2^_bootstrapping_= 0.7 ± 0.2, *p<*0.01) (see also Supplementary Table 3 for overall soil correlations among all variables analyzed).

Mean δ^13^C_foliar_ per site shows an inverse relationship with elevation (R^2^_bootstrapping_= 0.47 ± 0.1, *p<*0.001), but the photosynthetic pathway is the first order factor that determines the δ^13^C_foliar_ values (Supplementary Fig. 1b and Supplementary Table 2). *Tiquilia atacamensis, Jarava frigida, Parastrephia quadrangularis* and *Baccharis tola* are C3 plants and show an average δ^13^C_foliar_ of -26,2‰, -24.6‰, -22.9‰ and -21.9‰ respectively. The C4 shrub *Atriplex imbricata* shows δ^13^C_foliar_ values from a -17.0‰ to -13.9‰ (mean of -15.3‰). The δ^13^C values from the cacti *Maihueniopsis camachoi* (a CAM) ranges from -24.0‰ to -11.5‰ (mean -13.3‰).

We also examined the distribution of photosynthetic pathways across the TLT. The most abundant C_4_ plants is *Atriplex imbricata* although annual grasses (*Munroa, Bouteloua, Aristida*), occur from 3500 to 2900 m. during wet years. The CAM *Maihueniopsis camachoi* (Cactaceae) also grows from 4100 to 2700 m. The contribution of these C_4_ and CAM plants should predictably generate higher δ^13^C values in soil samples from those elevations (Supplementary Fig. 1).

SUPPLEMENTARY FIGURES

Supplementary Figure 1: δ^13^C from soils and plants.

Elevation versus a) Mean soil δ^13^C and b) Foliar δ^13^C values for species specific to given photosynthetic pathways across the TLT.

Supplementary Figure 2: Normalized foliar and soil δ^15^N values.

Boxplot comparing normalized foliar and soil δ^15^N values from the different vegetation belts.

SUPPLEMENTARY TABLES

Supplementary Table 1: Soil isotopes values for all soils analyzed (n:50)

| **Site** | **Isotope Laboratory** | **Elevation** | **δ^15^N** | **δ^13^C** | **%N** | **%C** | **C/N** | **Year** |
| --- | --- | --- | --- | --- | --- | --- | --- | --- |
| TLT01 | COIL | 4480 | 6.5 | -23.0 | 0.02 | 0.18 | 11.09 | 2012 |
| TLT01 | COIL | 4480 | 5.3 | -23.7 | 0.01 | 0.14 | 12.96 | 2012 |
| TLT02 | LABASI | 4370 | 8.0 | -23.8 | 0.02 | 0.23 | 10.29 | 2013 |
| TLT02 | LABASI | 4370 | 7.2 | -24.8 | 0.02 | 0.15 | 9.19 | 2012 |
| TLT03 | COIL | 4270 | 6.1 | -24.4 | 0.01 | 0.16 | 11.94 | 2012 |
| TLT03 | LABASI | 4270 | 7.2 | -24.5 | 0.02 | 0.18 | 11.02 | 2013 |
| TLT04 | COIL | 4174 | 6.5 | -24.1 | 0.02 | 0.25 | 11.79 | 2012 |
| TLT04 | LABASI | 4174 | 5.3 | -23.5 | 0.03 | 0.32 | 10.73 | 2013 |
| TLT05 | COIL | 4072 | 3.3 | -22.6 | 0.02 | 0.19 | 11.02 | 2011 |
| TLT05 | COIL | 4072 | 4.8 | -22.6 | 0.02 | 0.28 | 11.19 | 2011 |
| TLT06 | COIL | 3970 | 5.5 | -22.3 | 0.02 | 0.33 | 15.47 | 2012 |
| TLT06 | LABASI | 3970 | 8.1 | -22.1 | 0.02 | 0.24 | 12.13 | 2013 |
| TLT07 | COIL | 3870 | 8.4 | -23.1 | 0.01 | 0.20 | 13.76 | 2012 |
| TLT07 | COIL | 3870 | 7.1 | -22.0 | 0.04 | 0.49 | 12.70 | 2012 |
| TLT08 | COIL | 3870 | 8.1 | -22.2 | 0.02 | 0.24 | 11.99 | 2012 |
| TLT08 | LABASI | 3870 | 7.0 | -22.7 | 0.03 | 0.40 | 14.37 | 2013 |
| TLT09 | COIL | 3770 | 10.0 | -22.2 | 0.03 | 0.26 | 10.42 | 2012 |
| TLT09 | LABASI | 3770 | 8.2 | -23.2 | 0.02 | 0.27 | 10.65 | 2013 |
| TLT10 | COIL | 3670 | 8.5 | -21.8 | 0.02 | 0.19 | 10.82 | 2012 |
| TLT10 | LABASI | 3670 | 7.4 | -22.2 | 0.02 | 0.18 | 10.85 | 2013 |
| TLT11 | COIL | 3570 | 7.7 | -21.5 | 0.02 | 0.16 | 10.17 | 2012 |
| TLT11 | LABASI | 3570 | 11.3 | -20.8 | 0.02 | 0.14 | 8.17 | 2013 |
| TLT12 | COIL | 3470 | 8.7 | -19.4 | 0.02 | 0.19 | 9.71 | 2012 |
| TLT12 | LABASI | 3470 | 8.7 | -19.8 | 0.01 | 0.16 | 12.04 | 2013 |
| TLT13 | COIL | 3370 | 6.3 | -21.2 | 0.02 | 0.24 | 10.90 | 2012 |
| TLT13 | COIL | 3370 | 6.9 | -21.9 | 0.04 | 0.41 | 9.73 | 2012 |
| TLT14 | LABASI | 3270 | 10.3 | -16.8 | 0.03 | 0.22 | 7.78 | 2013 |
| TLT14 | COIL | 3270 | 10.8 | -19.0 | 0.02 | 0.18 | 8.69 | 2012 |
| TLT15 | COIL | 3170 | 9.1 | -17.7 | 0.03 | 0.18 | 6.72 | 2011 |
| TLT15 | COIL | 3170 | 7.6 | -20.2 | 0.02 | 0.11 | 6.10 | 2011 |
| TLT16 | COIL | 3070 | 11.1 | -18.7 | 0.01 | 0.13 | 8.64 | 2012 |
| TLT16 | LABASI | 3070 | 9.0 | -18.2 | 0.01 | 0.08 | 7.42 | 2013 |
| TLT17 | COIL | 2970 | 12.1 | -13.3 | 0.02 | 0.14 | 7.82 | 2012 |
| TLT17 | LABASI | 2970 | 8.0 | -15.4 | 0.02 | 0.13 | 6.85 | 2013 |
| TLT18 | COIL | 2870 | 9.6 | -19.1 | 0.01 | 0.07 | 6.74 | 2012 |
| TLT18 | LABASI | 2900 | 9.8 | -17.5 | 0.01 | 0.12 | 8.79 | 2013 |
| TLT19 | COIL | 2770 | 12.2 | -11.7 | 0.01 | 0.13 | 12.49 | 2012 |
| TLT19 | COIL | 2770 | 10.0 | -14.8 | 0.01 | 0.12 | 9.79 | 2012 |
| TLT20 | LABASI | 2700 | 9.2 | -7.0 | 0.01 | 0.12 | 21.61 | 2013 |
| TLT20 | LABASI | 2700 | 8.2 | -17.8 | 0.01 | 0.06 | 5.89 | 2013 |

Supplementary Table 2: Foliar isotope values for all plants analyzed (n:66).

| *Foliar* |  |  |  |  |  |  |  |
| --- | --- | --- | --- | --- | --- | --- | --- |
| **Site** | **Isotope Laboratory** | **Elevation** | **Taxa** | **Type** | **Photosynthetic pathway** | **δ^15^N** | **δ^13^C** |
| TLT01 | LABASI | 4480 | *Jarava frigida* | Grass | C3 | 1.7 | -24.0 |
| TLT01 | LABASI | 4480 | *Jarava frigida* | Grass | C3 | 2.6 | -25.2 |
| TLT02 | LABASI | 4370 | *Jarava frigida* | Grass | C3 | -0.6 | -25.0 |
| TLT02 | LABASI | 4370 | *Jarava frigida* | Grass | C3 | 3.9 | -25.1 |
| TLT03 | LABASI | 4270 | *Jarava frigida* | Grass | C3 | 3.5 | -27.0 |
| TLT04 | LABASI | 4174 | *Jarava frigida* | Grass | C3 | -0.3 | -25.0 |
| TLT05 | LABASI | 4072 | *Jarava frigida* | Grass | C3 | -1.0 | -25.0 |
| TLT05 | LABASI | 4072 | *Jarava frigida* | Grass | C3 | 0.3 | -22.8 |
| TLT06 | LABASI | 3970 | *Jarava frigida* | Grass | C3 | -1.2 | -22.2 |
| TLT06 | LABASI | 3970 | *Jarava frigida* | Grass | C3 | 5.5 | -26.6 |
| TLT07 | LABASI | 3870 | *Jarava frigida* | Grass | C3 | 2.8 | -24.1 |
| TLT07 | LABASI | 3870 | *Jarava frigida* | Grass | C3 | -2.0 | -23.8 |
| TLT02 | COIL | 4370 | *Parastrephia quadrangularis* | Shrub | C3 | 4.8 | -24.1 |
| TLT03 | COIL | 4270 | *Parastrephia quadrangularis* | Shrub | C3 | 2.9 | -24.0 |
| TLT04 | COIL | 4174 | *Parastrephia quadrangularis* | Shrub | C3 | 2.4 | -23.3 |
| TLT05 | COIL | 4072 | *Parastrephia quadrangularis* | Shrub | C3 | 5.8 | -25.0 |
| TLT06 | COIL | 3970 | *Parastrephia quadrangularis* | Shrub | C3 | 5.6 | -13.7 |
| TLT07 | COIL | 3870 | *Parastrephia quadrangularis* | Shrub | C3 | 2.9 | -22.7 |
| TLT08 | COIL | 3870 | *Parastrephia quadrangularis* | Shrub | C3 | 5.4 | -24.2 |
| TLT09 | COIL | 3770 | *Parastrephia quadrangularis* | Shrub | C3 | 5.3 | -26.4 |
| TLT05 | COIL | 4072 | *Baccharis tola* | Shrub | C3 | 0.3 | -24.9 |
| TLT06 | COIL | 3970 | *Baccharis tola* | Shrub | C3 | 2.7 | -20.3 |
| TLT06 | COIL | 3970 | *Baccharis tola* | Shrub | C3 | 5.0 | -21.9 |
| TLT07 | COIL | 3870 | *Baccharis tola* | Shrub | C3 | 4.4 | -21.4 |
| TLT07 | COIL | 3870 | *Baccharis tola* | Shrub | C3 | 5.4 | -21.8 |
| TLT08 | COIL | 3870 | *Baccharis tola* | Shrub | C3 | 1.8 | -20.4 |
| TLT08 | COIL | 3870 | *Baccharis tola* | Shrub | C3 | 7.9 | -19.3 |
| TLT10 | COIL | 3670 | *Baccharis tola* | Shrub | C3 | 5.4 | -21.6 |
| TLT10 | COIL | 3670 | *Baccharis tola* | Shrub | C3 | 4.3 | -21.6 |
| TLT10 | COIL | 3670 | *Baccharis tola* | Shrub | C3 | 7.5 | -24.1 |
| TLT12 | COIL | 3470 | *Baccharis tola* | Shrub | C3 | 3.6 | -21.1 |
| TLT12 | COIL | 3470 | *Baccharis tola* | Shrub | C3 | 3.7 | -23.8 |
| TLT12 | COIL | 3470 | *Atriplex imbricata* | Shrub | C4 | 3.7 | -16.0 |
| TLT12 | COIL | 3470 | *Atriplex imbricata* | Shrub | C4 | 8.2 | -15.0 |
| TLT13 | COIL | 3370 | *Atriplex imbricata* | Shrub | C4 | 8.8 | -15.6 |
| TLT13 | COIL | 3370 | *Atriplex imbricata* | Shrub | C4 | 7.9 | -14.6 |
| TLT14 | COIL | 3270 | *Atriplex imbricata* | Shrub | C4 | 8.4 | -15.5 |
| TLT14 | COIL | 3270 | *Atriplex imbricata* | Shrub | C4 | 7.5 | -14.4 |
| TLT15 | COIL | 3170 | *Atriplex imbricata* | Shrub | C4 | 5.2 | -17.0 |
| TLT15 | COIL | 3170 | *Atriplex imbricata* | Shrub | C4 | 7.7 | -14.3 |
| TLT16 | COIL | 3070 | *Atriplex imbricata* | Shrub | C4 | 4.0 | -15.6 |
| TLT16 | COIL | 3070 | *Atriplex imbricata* | Shrub | C4 | 7.1 | -14.1 |
| TLT17 | COIL | 2970 | *Atriplex imbricata* | Shrub | C4 | 3.3 | -16.2 |
| TLT17 | COIL | 2970 | *Atriplex imbricata* | Shrub | C4 | 3.8 | -13.9 |
| TLT18 | COIL | 2870 | *Atriplex imbricata* | Shrub | C4 | 5.4 | -16.7 |
| TLT18 | COIL | 2870 | *Atriplex imbricata* | Shrub | C4 | 6.3 | -14.8 |
| TLT07 | COIL | 3870 | *Maihueniopsis camachoi* | Cactus | CAM | 7.5 | -16.3 |
| TLT07 | COIL | 3870 | *Maihueniopsis camachoi* | Cactus * | CAM | 6.4 | -12.9 |
| TLT08 | COIL | 3870 | *Maihueniopsis camachoi* | Cactus * | CAM | 6.7 | -13.1 |
| TLT10 | COIL | 3670 | *Maihueniopsis camachoi* | Cactus | CAM | 5.6 | -12.9 |
| TLT10 | COIL | 3670 | *Maihueniopsis camachoi* | Cactus * | CAM | 7.4 | -12.5 |
| TLT11 | COIL | 3570 | *Maihueniopsis camachoi* | Cactus * | CAM | 5.1 | -11.5 |
| TLT12 | COIL | 3470 | *Maihueniopsis camachoi* | Cactus * | CAM | 6.7 | -12.1 |
| TLT13 | COIL | 3370 | *Maihueniopsis camachoi* | Cactus * | CAM | 5.4 | -11.8 |
| TLT14 | COIL | 3270 | *Maihueniopsis camachoi* | Cactus | CAM | 6.9 | -13.0 |
| TLT14 | COIL | 3270 | *Maihueniopsis camachoi* | Cactus * | CAM | 6.4 | -12.3 |
| TLT15 | COIL | 3170 | *Maihueniopsis camachoi* | Cactus * | CAM | 4.6 | -11.7 |
| TLT16 | COIL | 3070 | *Maihueniopsis camachoi* | Cactus * | CAM | 5.4 | -11.5 |
| TLT17 | COIL | 2970 | *Maihueniopsis camachoi* | Cactus * | CAM | 5.8 | -11.7 |
| TLT19 | COIL | 2770 | *Maihueniopsis camachoi* | Cactus * | CAM | 7.1 | -12.5 |
| TLT20 | COIL | 2670 | *Maihueniopsis camachoi* | Cactus | CAM | 4.4 | -24.0 |
| TLT20 | COIL | 2670 | *Maihueniopsis camachoi* | Cactus * | CAM | 3.7 | -12.9 |
| TLT14 | LABASI | 3270 | *Tiquilia atacamensis* | Sub-shrub | C3 | 6.6 | -25.8 |
| TLT15 | LABASI | 3170 | *Tiquilia atacamensis* | Sub-shrub | C3 | 7.0 | -26.6 |
| TLT15 | LABASI | 3170 | *Tiquilia atacamensis* | Sub-shrub | C3 | 6.1 | -25.3 |
| TLT18 | LABASI | 2870 | *Tiquilia atacamensis* | Sub-shrub | C3 | 6.3 | -27.1 |
| All plant samples were collected during the wet (April) 2012 season | | | |  |  |  |  |
| * These are seeds samples, to contrast with stems in *Maihueniopsis camachoi* | | | | |  |  |  |

Supplementary Table 3: Soil isotopes values at different depths.

| **Elavation and Depht** | **δ^15^N** | **δ^13^C** | **% N** | **% C** | **C/N** |
| --- | --- | --- | --- | --- | --- |
| 4300 m (0 cm depth) | 7.2 | -24.5 | 0.02 | 0.18 | 11.0 |
| 4300 m (25 cm depth) | 8.0 | -22.1 | 0.03 | 0.25 | 9.51 |
| 4300 m (50 cm depth) | 8.0 | -21.6 | 0.02 | 0.19 | 10.7 |
|  |  |  |  |  |  |
| 3600 m (0 cm depth) | 11.3 | -20.8 | 0.02 | 0.14 | 8.17 |
| 3600 m (25 cm depth) | 10.0 | -18.8 | 0.03 | 0.27 | 9.26 |
| 3600 m (50 cm depth) | 10.2 | -19.1 | 0.02 | 0.14 | 8.74 |
|  |  |  |  |  |  |
| 2900 m (0 cm depth) | 9.8 | -17.5 | 0.01 | 0.12 | 8.79 |
| 2900 m (25 cm depth) | 10.2 | -20.3 | 0.01 | 0.06 | 7.59 |
| 2900 m (50 cm depth) | 10.0 | -20.4 | 0.01 | 0.07 | 9.6 |
| * All these samples were collected during April 2013 and were | | | | | |
| analized at the LABASI (Isotope laboratory) | | | |  |  |
